# Neoplasia and infectious disease in the critically endangered species *Crax alberti*

**DOI:** 10.29374/2527-2179.bjvm007525

**Published:** 2026-06-12

**Authors:** Santiago Duque-Arias, Natalia Maria Granda-Orozco, Mónica Montejo-Casteñeda

**Affiliations:** 1 1Grupo de Investigación GINVER, Facultad de Medicina Veterinaria, Corporación Universitaria Remington, Medellín, Antioquia, Colombia.; 2 Parque de la Coservación, Medellín, Antioquia, Colombia.

**Keywords:** carcinoma, Mycoplasma, pathology, zoo animals, wildlife, carcinoma, Mycoplasma, patologia, animais de zoológico, fauna silvestre

## Abstract

The following report presents the results of clinical, pathological and, histopathological analyses of two individuals of *Crax alberti*, a critically endangered species. One individual was diagnosed with cholangiocellular carcinoma with concurrent respiratory involvement, while the other presented pneumonia associated with *Mycoplasma* spp., with secondary renal involvement. In captive conditions, increased longevity and close contact between individuals may favor the occurrence of infectious and neoplastic conditions, as observed in these cases.

## Resumo

O presente relato de caso apresenta os resultados das análises clínicas, patológicas e histopatológicas de dois indivíduos de *Crax alberti*, uma espécie criticamente ameaçada de extinção. Um indivíduo foi diagnosticado com carcinoma colangiocelular com envolvimento respiratório concomitante, enquanto o outro apresentou pneumonia associada a *Mycoplasma* spp., com envolvimento renal secundário. Em condições de cativeiro, o aumento da longevidade e o contato próximo entre indivíduos podem favorecer a ocorrência de condições infecciosas e neoplásicas, como observado nestes casos.

## Introduction

*Crax alberti*, or Blue-billed curassow, is a bird belonging to the Cracidae family that is critically endangered due to human disturbance, habitat loss, hunting, and wildlife trafficking (Valencia et al., 2023). It is endemic to the central mountain range of the Andes in Antioquia, Colombia, with few records available (Ochoa-Quintero et al., 2005).

Many endangered species are kept in captivity as part of conservation programs to increase their population and preserve their genetic material (Fatima, 2024). Under these conditions, life expectancy is often extended, which may increase the incidence of neoplasms and infectious diseases, the latter being favoured by high population density (Duque-Arias et al., 2025).

Reports of pathologies in animals under human care highlight the occurrence of respiratory diseases in birds, mammals and reptiles (González-R et al., 2015), as well as neoplasms of undetermined cell lineage (Pesavento et al., 2018). The aim of this report is to describe the clinical and pathological findings in two *Crax alberti* individuals that died in captivity in Medellín, Colombia, with diagnoses of pneumonia associated with *Mycoplasma* spp. and hepatic cholangiocellular carcinoma.

### Case report

#### Case 1 (Curassow 1)

**Clinical findings.** Curassow 1, a 23-year-old male, was hospitalized for anorexia and poor physiological condition. The haemoleukogram revealed lymphopenia. Tube feeding was initiated, however, the bird regurgitated. Nebulization was performed after the onset of rales. After nine days, a new blood test revealed moderate heterophilia, mild hypoalbuminaemia, moderate hyperuricaemia, and a marked increase in aspartate aminotransferase levels. Coelomic ultrasound revealed a hepatic mass with an infiltrative appearance. Given the poor prognosis, humane euthanasia was performed.

**Figure 1:**
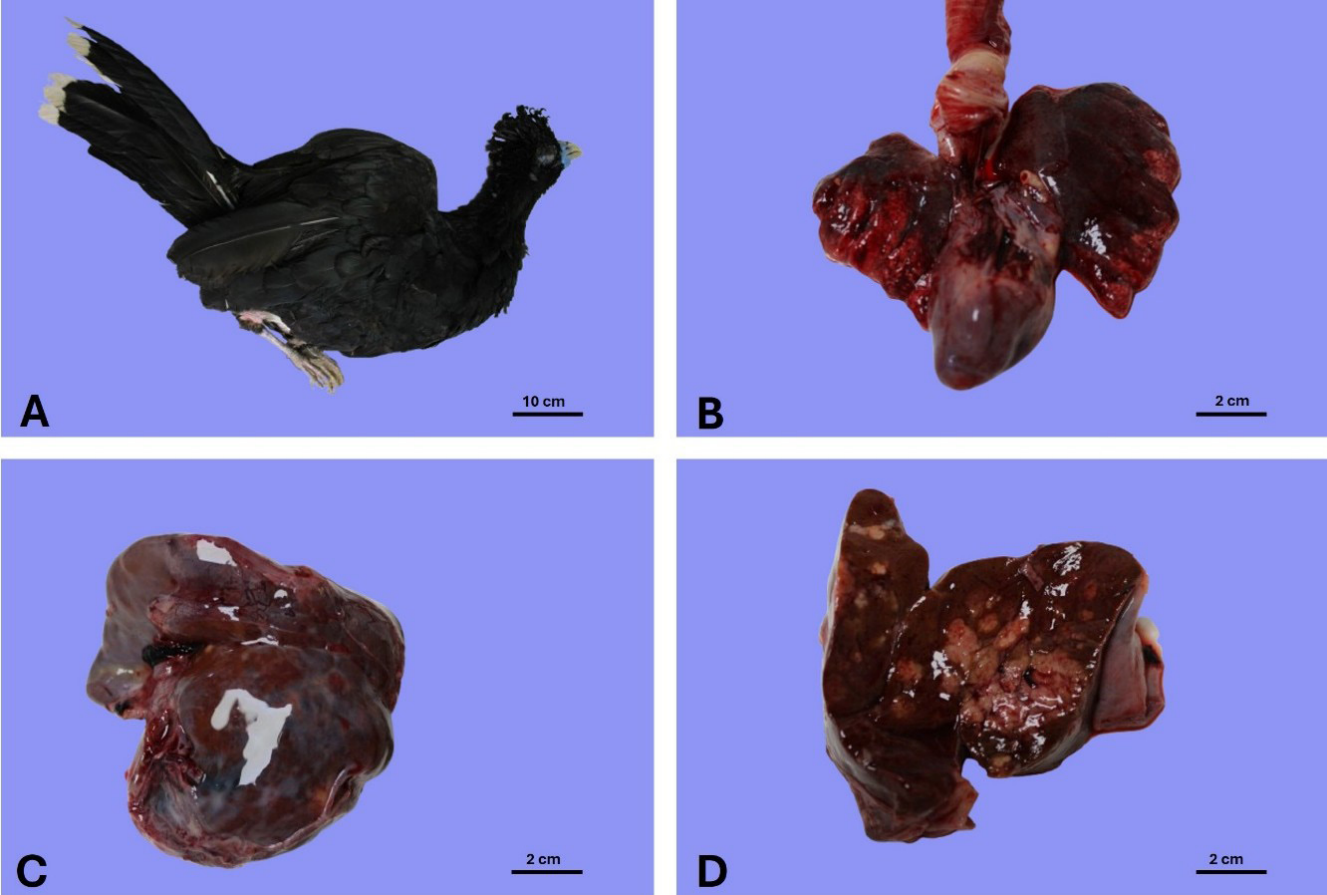
*Gross pathological findings of Curassow 1*.

*Note.* (A) External view of Curassow 1. (B) Lungs with cranial hepatization, with increased consolidation and dark red discoloration. (C) Liver with marked hepatomegaly and multifocal to coalescing whitish areas on the capsular surface. (D) Sectioned liver revealing a central mass with an infiltrative appearance and multiple white–pink nodular foci distributed throughout the parenchyma.

**Gross findings.** At necropsy, Curassow 1 (Figure 1) exhibited discharge from the nostrils and oral cavity, a body condition score of 2/5, fecal staining, and abdominal petechiae. Upon skin removal, fat necrosis and subcutaneous emphysema were observed. The trachea contained fibrin, purulent exudate, and necrotic material, and the lungs showed moderate cranial hepatization (Figure 1B). The coelomic cavity had a reddish to yellow discoloration, and the translucent fluid within it had a sweet odour. The bird presented severe hepatomegaly with white-pink foci in the parenchyma (Figures 11D).

**Figure 2:**
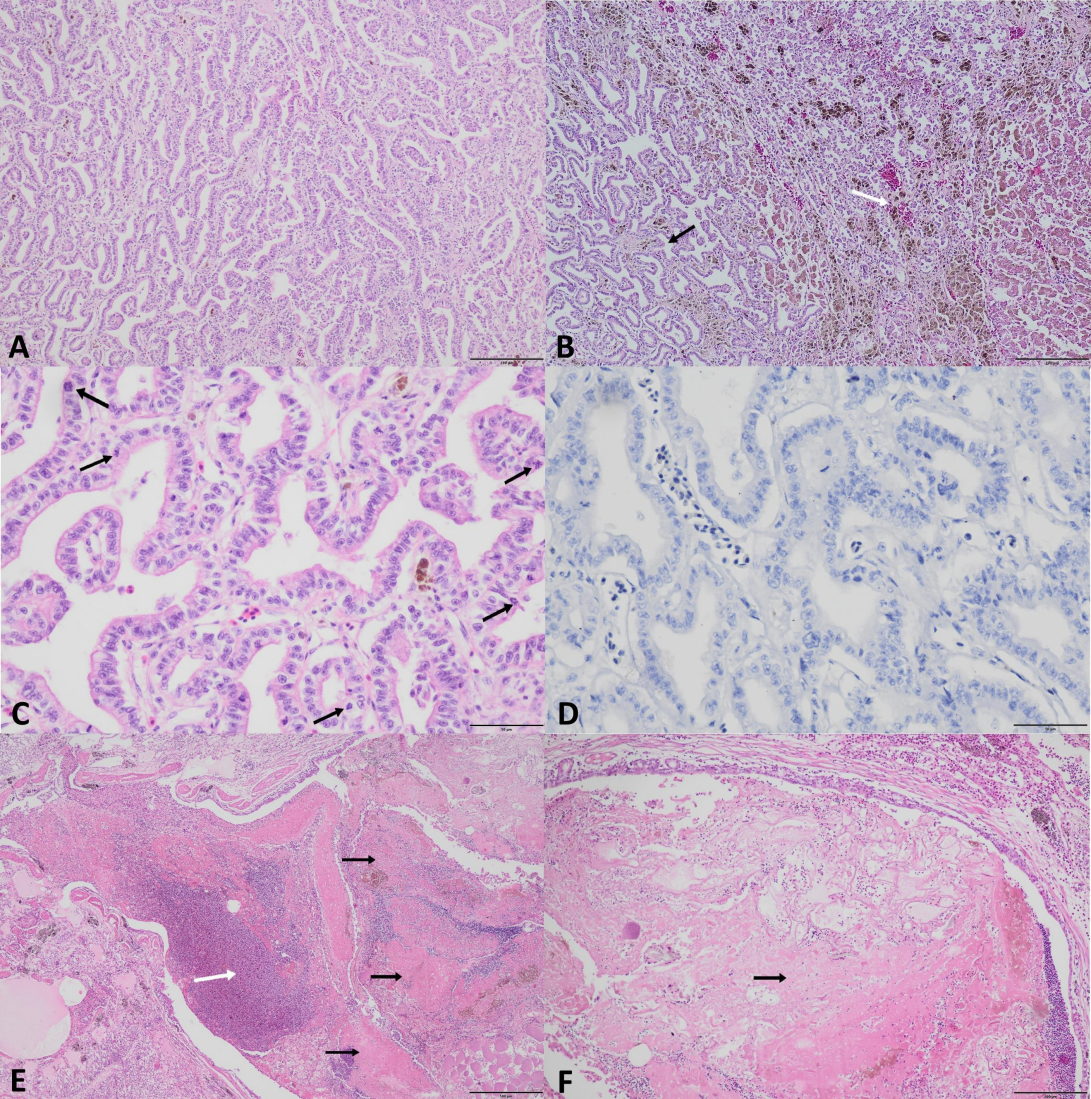
*Histopathological findings in Curassow 1*.

*Note.* (A) Liver (H&E). Cholangiocellular carcinoma. (B) Liver (H&E). Transition between neoplastic tissue (black arrow) and tissue free of neoplasia, with severe intrahepatocytic cholestasis. (C) Liver (H&E). Cholangiocellular carcinoma with abundant mitotic cells (black arrows). (D) Ki-67-negative immunohistochemistry. (E) Lung (H&E). Foci of necrosis (black arrows) with leukocyte infiltration (white arrow). (F) Lung (H&E). Luminal necrotic exudate in the airway (black arrow).

**Histopathological findings.** The main histopathological lesions of Curassow 1 included a hepatic epithelial neoplasm (Figure 2AC) infiltrating the stroma and adjacent parenchyma, arranged in duct-like structures lined by simple cuboidal to columnar epithelium with apical microvilli. Some neoplastic cells were multinucleated and exhibited karyomegaly and moderate poikiloanisokaryosis. A total of 17 mitotic figures were observed in 10 high-power fields (400x), along with a moderate lymphocytic infiltrate. Immunohistochemistry for Ki-67 was performed to evaluate cell proliferation using a commercially available monoclonal anti-Ki-67 antibody and a polymer-based detection system (Master Polymer Plus Detection System, Vitro Master Diagnóstica®, Spain), yielding a negative result (Figure 2D). In the lung, there was moderate consolidation with oedema, mononuclear leukocyte infiltration, and vascular congestion (Figure 2E), as well as thickened bronchi containing necrotic material, erythrocytes, and leukocytes within the lumen (Figure 2F).

#### Case 2 (Curassow 2)

**Figure 3:**
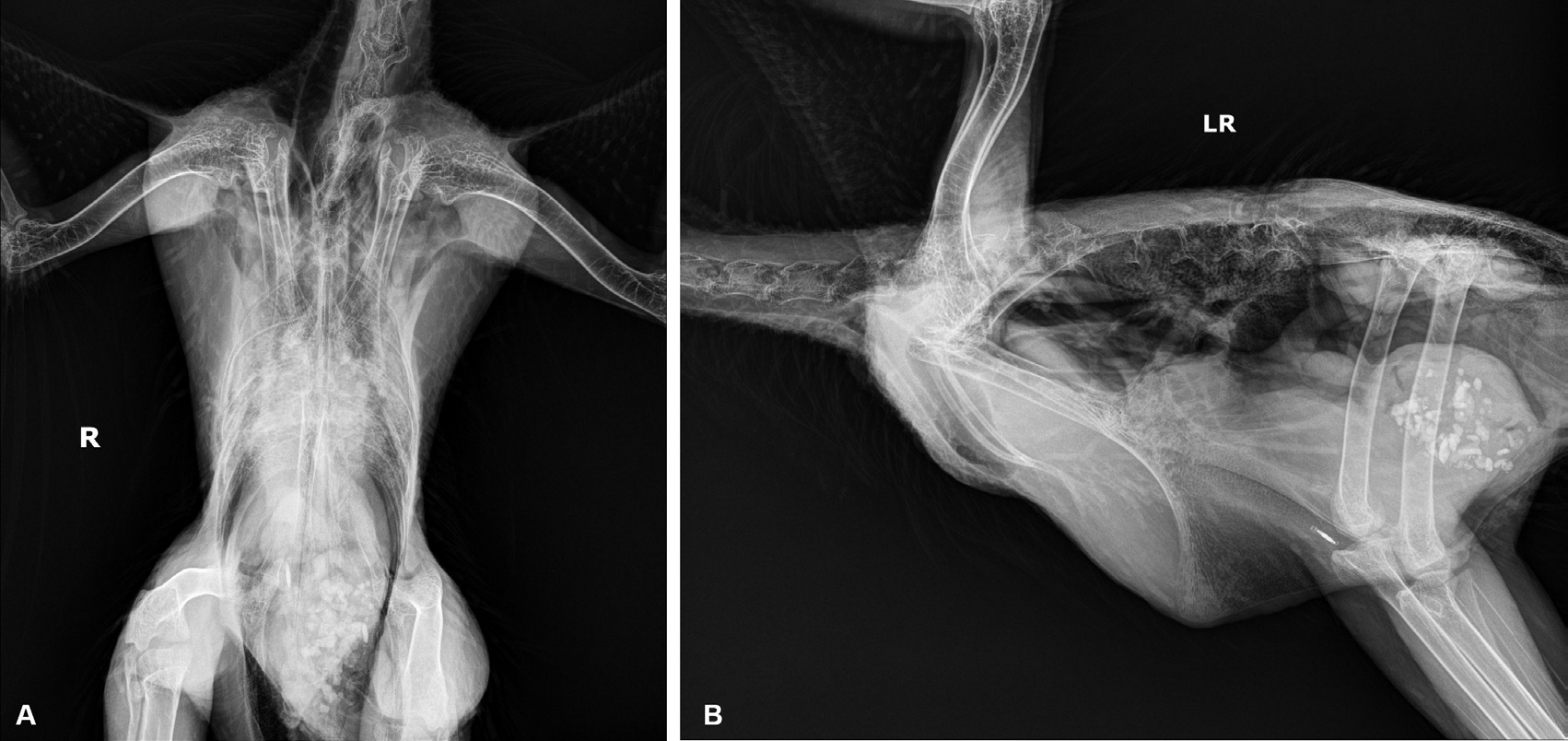
*Radiographic findings in Curassow 2*.

*Note.* (A) Ventrodorsal and (B) right lateral radiographic views. The cardiothoracic index is approximately 63%, with the trachea in normal position and well-defined margins. Cardiac, hepatic, renal, and gonadal silhouettes are preserved without evidence of increased radiodensity. The lungs show a diffuse bronchointerstitial pattern with a mottled appearance and radiodense thickening of the bronchial walls, consistent with moderate diffuse pneumonitis. Air sacs appear adequately aerated.

**Clinical findings.** Curassow 2, a 21-year-old male, was hospitalized due to reduced food intake. Treatment for coccidiosis was initiated based on stool analysis. Subsequently, the bird developed diarrhoea and rales, for which intravenous fluid therapy and nebulization were administered. A coelomic radiograph revealed moderate diffuse pneumonia (Figure 3), and a tracheobronchial lavage culture confirmed the presence of *Escherichia coli*. Antibiotic therapy was administered according to antimicrobial susceptibility testing; however, the bird did not respond to treatment and developed orthopnea, dying eight days after hospitalization.

**Figure 4:**
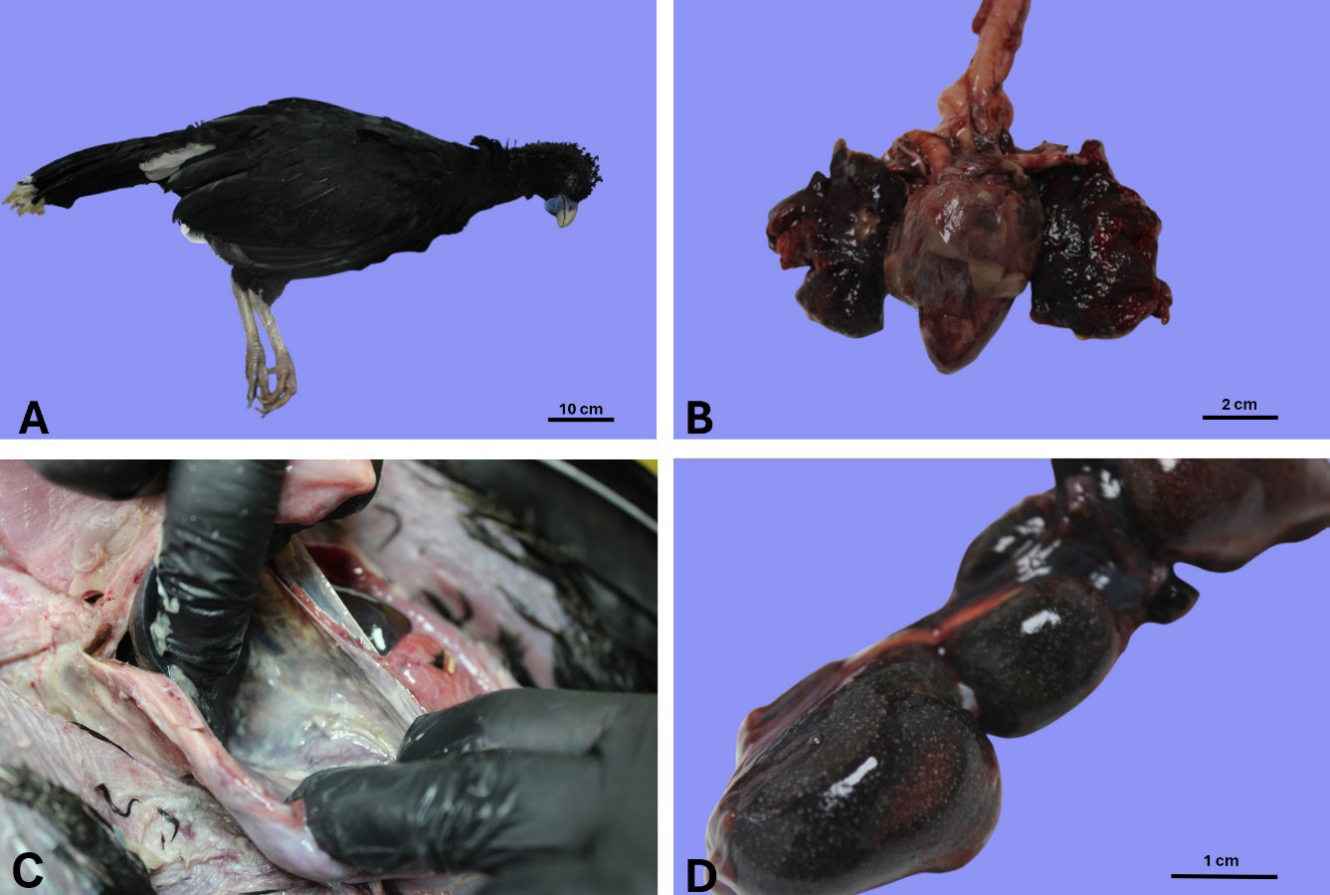
*Gross pathological findings of Curassow 2*.

*Note.* (A) External view of Curassow 2. (B) Lungs with marked dark discoloration and consolidation, associated with a thickened, whitish pericardium. (C) Coelomic cavity showing fibrinous adhesions involving visceral organs. (D) Kidneys exhibiting nephromegaly with multifocal whitish foci distributed across the capsular surface.

**Gross findings.** At necropsy, Curassow 2 (Figure 4) exhibited discharge from the nostrils and oral cavity. The trachea contained hyaline mucus, and the lungs showed dark discoloration (Figure 4B). The air sacs were thickened and whitish, with adhesions within the coelomic cavity involving the lungs, pericardium, and cranial portion of the liver, along with deposits of dark brown fluid (Figure 4C). The pericardium was thickened, whitish and distended with fluid, whereas the hepatic capsule was thickened in its cranial portion. The large intestine showed petechial haemorrhage. The kidneys had an irregular surface, nephromegaly and multifocal whitish foci in the capsule and parenchyma (Figure 4D). Additionally, during necropsy, a swab of the pulmonary content was collected for molecular analysis targeting *Chlamydia* spp. and *Mycoplasma* spp. by real-time PCR (qPCR), which yielded a positive result for *Mycoplasma* spp., with a bacterial load greater than 10,000 copies/µL (laboratory results are provided in the Supplementary Material).

**Figure 5:**
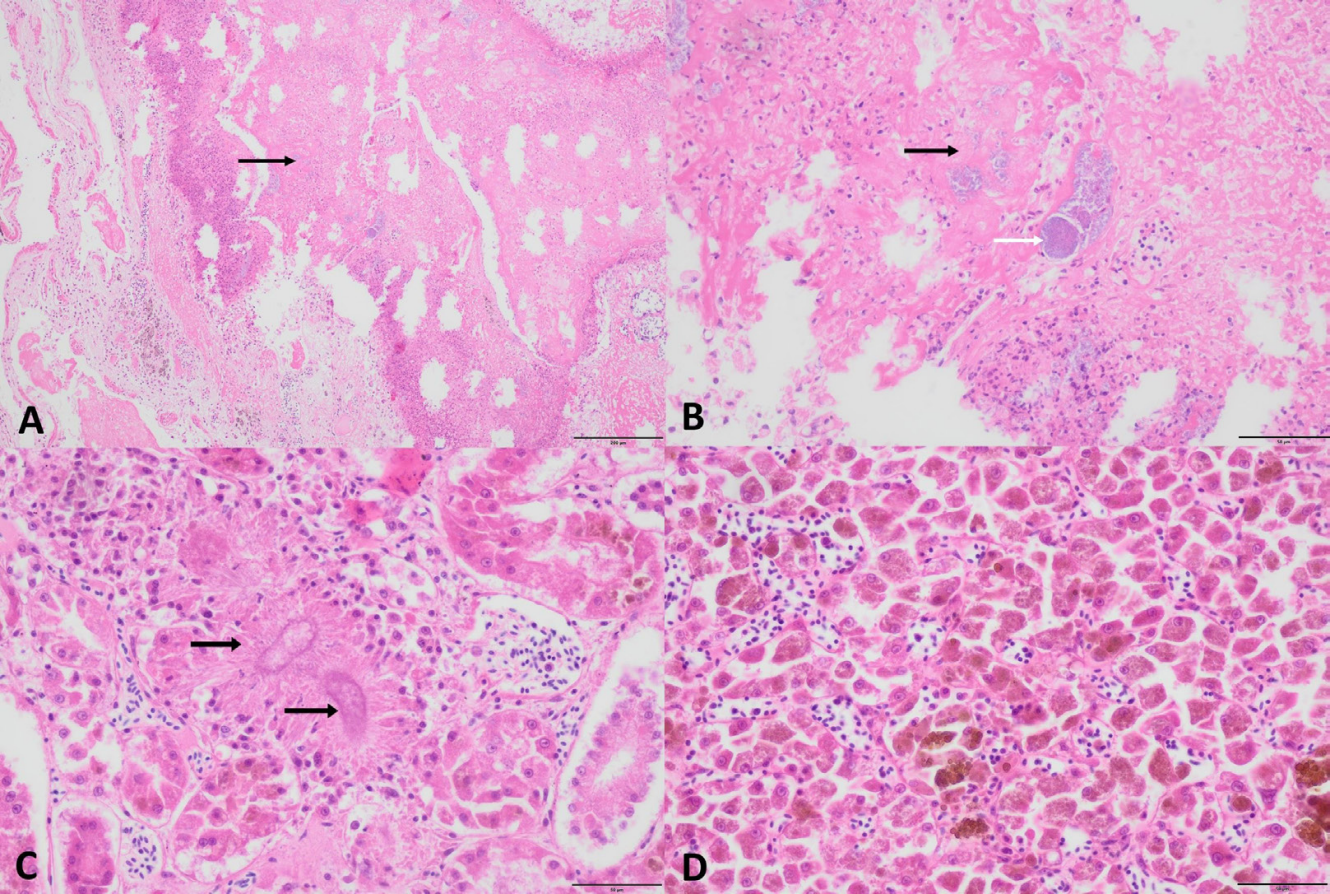
*Histopathological findings in Curassow 2*.

*Note.* (A) Lung (H&E). Necrosis (black arrow). (B) Lung (H&E). Necrosis (black arrow) and bacterial colonies (white arrow). (C) Kidney (H&E). Uric acid deposits (black arrows). (D) Liver (H&E). Intrahepathocytic cholestasis and sinusoidal congestion.

**Histopathological findings.** The main histopathological lesions of Curassow 2 were primarily located in the lung, with foci of necrosis, abundant fibrin deposits and bacterial colonies (Figure 5AB). Around the necrotic areas, multinucleated epithelioid and giant cells were observed, along with marked vascular congestion in air capillaries and large vessels. Anthracosis was also observed. The renal parenchyma presented urate deposits, glomerular atrophy and moderate tubular epithelial degeneration. The liver showed moderate degeneration (Figure 5D). In the heart, histiocytes and bacterial colonies were identified in the epicardium. The spleen showed severe lymphoid depletion and marked histiocytic proliferation. In the pancreas, foci of necrosis similar to those observed in the lungs were also identified.

## Discussion

**Figure 6:**
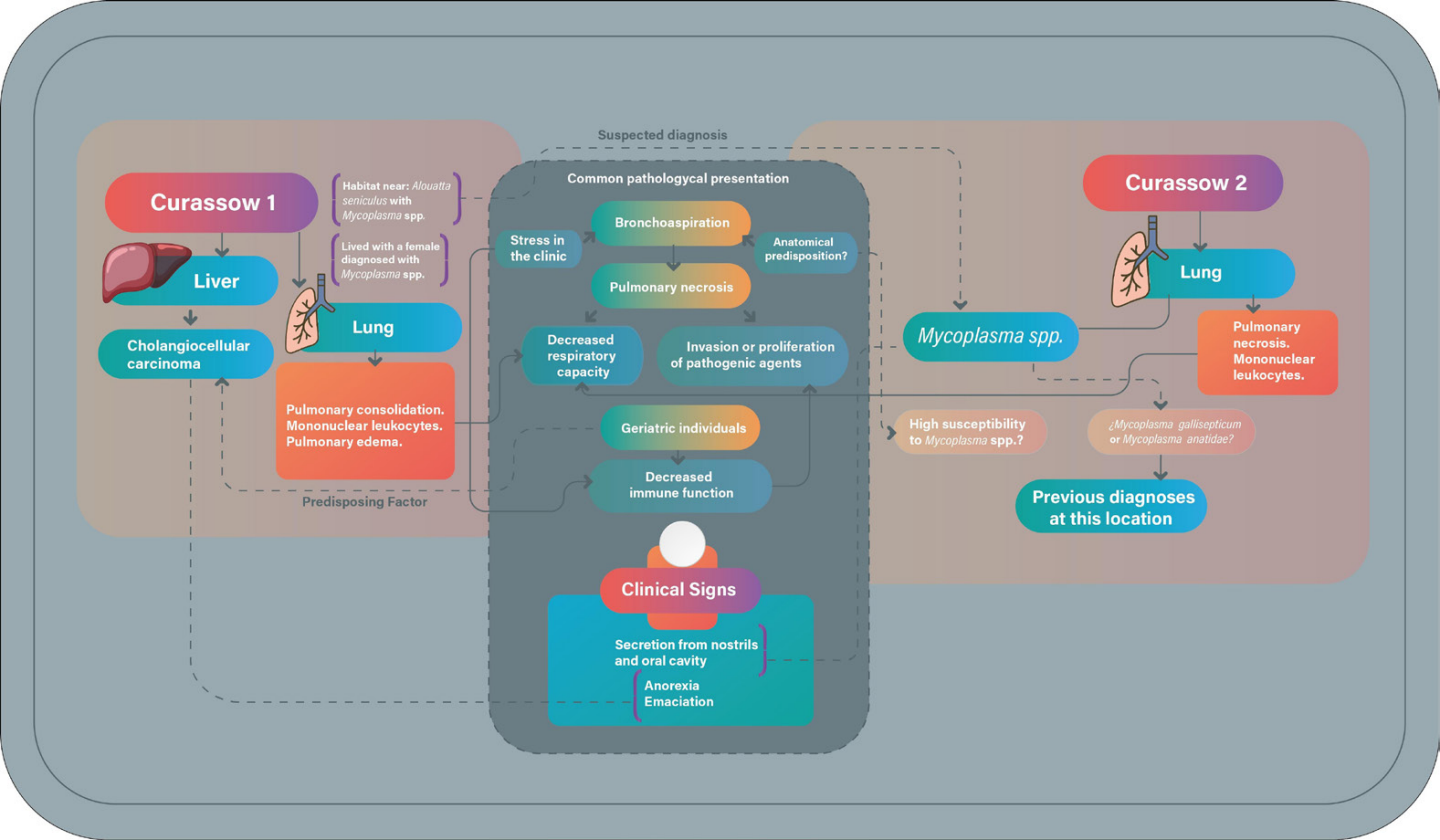
*Diagram and analysis of the pathological findings of two Crax alberti individuals*.

*Note.* Schematic representation of the main pathological findings and proposed mechanisms in both *Crax alberti* individuals. The diagram summarizes the association between respiratory lesions, and the detection of *Mycoplasma* spp., as well as the presence of cholangiocellular carcinoma in Curassow 1. It also illustrates potential contributing factors such as advanced age, stress associated with captivity, and possible anatomical predisposition, which may have influenced disease progression and clinical presentation.

This is the first report describing anatomical and histopathological findings in two *C. alberti* individuals that died under conservation conditions. These findings may contribute to conservation efforts and to the clinical management of individuals presenting similar signs in captivity. To our knowledge, this is the first report evaluating Ki-67 in cracids. Although widely used in veterinary oncology, particularly in domestic mammals, no immunoreactivity was observed in this case, and its applicability in wild avian species remains uncertain. Figure 6 summarizes the main pathological findings and the hypotheses proposed in this study.

Although both individuals shared certain clinical and pathological features, their distinct etiologies and the limited number of cases suggest that these observations should be interpreted with caution. Both individuals were hospitalized for several days prior to death and presented clinical signs such as emaciation, anorexia, and discharge from the nostrils and oral cavity, which may be consistent with respiratory involvement, even in Curassow 1, whose euthanasia was primarily due to neoplasia. Notably, Curassow 1 was housed in proximity to an *Alouatta seniculus* individual positive for *Mycoplasma* spp. and had previously cohabited with a female diagnosed with the same infection (laboratory information available in Duque-Arias, 2025), suggesting that transmission may have occurred via aerosols over varying distances, environmental exposure, or direct contact with infected birds, among other possible routes (Mugunthan et al., 2023).

In Curassow 2, the presence of diffuse pneumonia, fibrin deposition, and bacterial colonies, together with the positive qPCR result for *Mycoplasma* spp., may support the involvement of a respiratory infectious process. In addition, both *Mycoplasma* spp. (detected by qPCR) and *Escherichia coli* (identified by culture) were present. In avian species, respiratory diseases are frequently multifactorial, and co-infections involving *Mycoplasma* spp. and *E. coli* have been widely reported (Samy & Naguib, 2018; Gowthaman et al., 2019). *Mycoplasma* spp. are recognized as primary respiratory pathogens that can impair mucosal defenses and modulate the immune response, thereby facilitating secondary bacterial infections such as colibacillosis (Samy & Naguib, 2018). In this context, the presence of *E. coli* may represent a secondary or opportunistic infection. However, given the limitations of the present study, the specific role and interaction of these agents cannot be definitively established.

*Mycoplasma* infections in birds are characterized by a wide host range and the ability to infect multiple avian taxa beyond their traditional galliform hosts, with evidence of exposure reported in numerous wild bird species across different families (Dhondt et al., 2014; Sawicka-Durkalec et al., 2021). This may support the plausibility of cross-species transmission in mixed-species environments such as zoological collections. In captive or rehabilitated wild birds, exposure to *Mycoplasma* spp. has been associated with conditions such as stress, overcrowding, and immunosuppression, which can facilitate infection and transmission (Marín-Villa et al., 2025). These factors may be relevant in captive wildlife settings and could have contributed to the clinical presentation observed in these cases. In Curassow 2, although *Mycoplasma* spp. infection was confirmed, species-level characterization was not achieved. However, *M. gallisepticum* and *M. anatidae* have been previously identified in the zoological collection, suggesting that these or related *Mycoplasma* species may be present in the area, although their role in these cases remains uncertain.

Aspiration during treatment was considered a possible contributing factor that may have influenced pulmonary involvement, particularly in Curassow 2. We hypothesize that this susceptibility could be influenced by anatomical features of the species, particularly the more caudally positioned glottis observed in these individuals (as shown in the necropsy macrophotographs provided in the Supplementary Material), together with the characteristic tracheal curvature of cracids (Amadon, 1978), which may increase the risk of aspiration compared to other avian species, although this remains speculative and requires further investigation. The resulting necrosis may facilitate the invasion and proliferation of pathogens (Košutová & Mikolka, 2021).

Both individuals were geriatric, suggesting age-related immunocompromise (Dhabhar, 2014), potentially exacerbated by stress associated with captivity (Fischer & Romero, 2019; Duque-Arias et al., 2025). This condition may have favoured the invasion and proliferation of pathogens. Regarding the hepatic neoplasm, primary liver tumors are considered uncommon in avian species, and the available information on their biological behavior, prognosis, and treatment remains limited, with most cases being identified *post mortem* (Reavill, 2004; Szweda et al., 2011). This scarcity of data underscores the importance of individual case reports in improving current knowledge. Furthermore, advanced age has been described as a risk factor for cholangiocarcinoma, a neoplasm reported in geriatric wildlife, which is frequently associated with the accumulation of fluid in the coelomic cavity, as observed in this case (Miranda et al., 2015; Lindemann et al., 2017).

Finally, this report has several limitations that should be acknowledged. It is based on only two cases, which restricts the ability to draw generalizable conclusions. Although clinical, pathological, and molecular findings were integrated, diagnostic limitations remain, including the lack of species-level characterization of *Mycoplasma* spp., the inherent constraints of *post-mortem* analyses, and the limited validation of immunohistochemical markers such as Ki-67 in wild avian species. Moreover, due to the descriptive nature of this report and the presence of distinct underlying etiologies in each case, causal relationships between the observed lesions and the identified pathogens cannot be established. These findings should therefore be interpreted as exploratory and contribute primarily to the documentation of pathological conditions in this critically endangered species.

## Conclusions

In the present cases, advanced age and captivity-related stress may have contributed to immunosuppression, which may have influenced the occurrence of infectious and neoplastic conditions in these individuals.

Respiratory lesions were observed in both individuals, although associated with different underlying conditions. In one case, *Mycoplasma* spp. was detected by molecular analysis. While these findings suggest a possible involvement of this pathogen, its specific role in the observed pathology cannot be established.
